# Draft Genome Sequences of Four Genetically Distinct Human Isolates of *Streptococcus dysgalactiae* subsp. *equisimilis*

**DOI:** 10.1128/genomeA.01139-15

**Published:** 2015-10-01

**Authors:** Caitlin Evers, Khushali Patel, Varduhi Petrosyan, Clay Morrison, Viju Varghese, Randy A. Chu, Aymen Baig, Erika J. Thompson, Michael Chase, Peter C. Hu, Awdhesh Kalia

**Affiliations:** a Graduate Program in Diagnostic Genetics, School of Health Professions, The University of Texas MD Anderson Cancer Center, Houston, Texas, USA; b Roche Diagnostics Corporation, Indianapolis, Indiana, USA; c Sequencing and Microarray Facility, The University of Texas MD Anderson Cancer Center, Houston, Texas, USA; d Molecular Genetic Technology Program, School of Health Professions, The University of Texas MD Anderson Cancer Center, Houston, Texas, USA

## Abstract

β-Hemolytic group C and group G streptococci (GCS-GGS; *Streptococcus dysgalactiae* subsp. *equisimilis*) emerged as human pathogens in the late 1970s. We report here the draft genome sequences of four genetically distinct human strains of GCS-GGS isolated between the 1960s and 1980s. Comparative analysis of these genomes may provide a deeper understanding of GCS-GGS genome and virulence evolution.

## GENOME ANNOUNCEMENT

Large-colony-forming β-hemolytic isolates of Lancefield group C and group G Streptococci (GCS-GGS) identified as *Streptococcus dysgalactiae* subsp. *equisimilis* can infect humans and other mammals (1, 2). GCS and GGS emerged as human pathogens in the late 1970s and early 1980s and now approximate or surpass group A streptococci (GAS) as the predominant cause of invasive β-hemolytic streptococcal infection (3–5). The transfer of genes from GAS into GCS-GGS genomes via horizontal gene transfer (HGT) is common and ostensibly the most parsimonious explanation for the emergence of GCS-GGS as human pathogens (1, 6, 7). The dynamics of HGT between GAS and GCS-GGS are complicated. Some HGT events are asymmetric (aHGT), which can further be categorized as additive- or replacing-type aHGT (7); other HGT events are more typical and involve homologous recombination among orthologs, resulting in gene mosaics (8, 9). The mechanisms of replacing-type aHGT remain elusive. It is unclear (i) how replacing-type aHGT may shape diversity in the global GCS-GGS gene pool, (ii) whether aHGT dynamics differ spatially and temporally, and (iii) whether aHGT alone can account for the emergence of GCS-GGS as human pathogens.

Here, we report the draft genome sequences of four GCS-GGS isolates (Table 1). Lancefield groups of all isolates were determined by serotyping. High-quality genomic DNA was extracted using a previously described method (10) modified to include mutanolysin. Isolates were confirmed to be *S. dysgalactiae* subsp. *equisimilis* with 16S rRNA sequencing (GenBank accession numbers KP972460 to KP972463), and the genetic relatedness of the GCS-GGS isolates was determined via multilocus sequence typing (MLST) (GenBank accession numbers KT347549 to KT347565) (Table 1) (11, 12). Whole-genome shotgun sequencing was done with the Roche 454 GS Jr+ system. *De novo* assembly was performed with Newbler version 3.0 using default settings; contigs <200 bp were not included. The genome statistics are listed in Table 1.

**TABLE 1:** Strain descriptions and draft genome statistics

Strain	Serotype/yr	Disease	MLST genotype	Accession no.	No. of contigs >200 bp	Contig N50 (Kb)	Average coverage (×)	Estimated genome size (Mb)
UT-5345	C/1983	Bacteremia	ST-53	LAKV00000000	91	50.369	24	2.2
UT-SS1069	C/1974	Unknown	ST-3	LAKS00000000	86	52	16	2.01
UT-5354	G/1980s	Bacteremia	ND^a^	LAKU00000000	75	75.47	21	2.07
UT-SS957	C/1969	Unknown	ST-51	LAKT00000000	58	91.808	50	2.03

a ND, not determined; harbors a unique *xpt* allele not present in the *S. dysgalactiae* subsp. *equisimilis* MLST database and 99% identical to allele *xpt28* from GCS-GGS and 99% identical to allele *xpt29* from GAS.

(This work was presented in part at the 115th General Meeting of the American Society for Microbiology, New Orleans, LA, 30 May to 2 June 2015.)

Assembled genomes were annotated using NCBI PGAP version 2.1 (rev. 462191) (http://www.ncbi.nlm.nih.gov/genome/annotation_prok/) and RASTtk pipelines (13) in conjunction with Blast2GO (14). The 16S rRNA sequences in the draft genome were identified using RNAmmer version 1.2 (15). Automated MLST using the draft genomes was performed with MLST version 1.8 (16). The pre- and postgenome sequencing of 16S rRNA and MLST sequence data were 100% concordant and confirmed the lack of any contaminating DNA in the genomic DNA preparations or in sequencing libraries.

### Nucleotide sequence accession numbers.

The draft genome sequences have been deposited as whole-genome shotgun projects at DDBJ/EMBL/GenBank under the accession numbers listed in Table 1. The versions described in this paper are the first versions.
